# A Rare Case of a Cervical Thymic Cyst Presenting in Adulthood

**DOI:** 10.1155/2020/4059530

**Published:** 2020-08-06

**Authors:** Blaine D. Smith, Michael H. Schild, Xiaoyin “Sara” Jiang, Russel R. Kahmke

**Affiliations:** ^1^Department of Head and Neck Surgery & Communication Sciences, Duke University, Durham, NC, USA; ^2^Department of Pathology, Duke University, Durham, NC, USA

## Abstract

The cervical thymic cyst (CTC) is a rare, benign neck mass that most commonly presents in the pediatric population. These entities can occur anywhere along the normal path of descent of the thymus from the mandible to the sternal notch, and extension into the mediastinum has been observed. The presentation of these masses is often characterized by a painless, enlarging neck mass in a child during the first decade of life. Although most patients are asymptomatic, abutment of the cyst against local structures has led to a variety of presentations including respiratory distress. These rare lesions are noted to have a male predominance and most commonly present on the left side of the neck. We present the rare case of a 19-year-old male who presented with a left-sided painless, cystic neck mass. He underwent a computed tomography scan of the neck which showed a large cystic mass in the left neck deep to the sternocleidomastoid muscle. Preoperatively, the diagnosis of an infected third branchial cyst was favored. The lesion was completely excised in the operating room. Final pathology was consistent with a CTC. The CTC is an uncommon benign process that often presents as an asymptomatic cystic neck mass. Knowledge of the clinical presentation, diagnostic process, and treatment of these rare lesions is essential for the Otolaryngologist.

## 1. Introduction

Cystic neck masses represent a diverse clinical entity ranging from congenital cysts to metastatic malignant disease. Out of the broad range of benign cystic neck masses, the majority represent infected congenital neck masses such as branchial cleft or thyroglossal duct cysts. The cervical thymic cyst is an extremely rare cystic neck mass [1, 2]. Of the lesions that have been described, the majority occur in children in the first decade of life after the age of two. The presentation of these lesions is often with a painless neck mass. Although most patients are asymptomatic, respiratory complications have been described. We present the unique case of an adult patient with a newly diagnosed cervical thymic cyst.

## 2. Case Presentation

**Figure 1:**
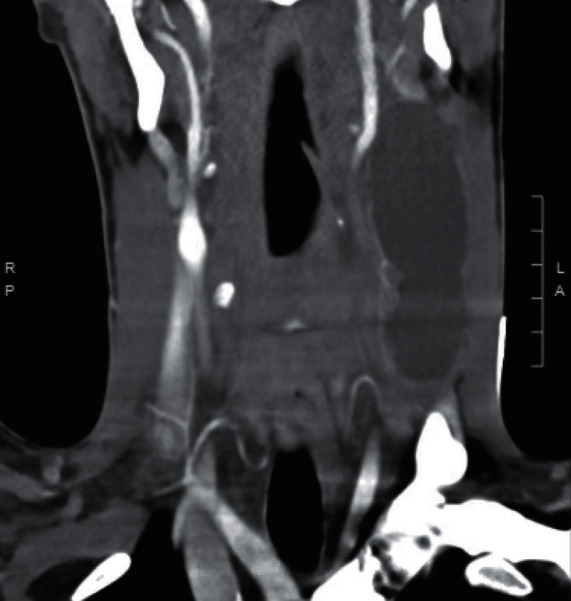
Computed tomography scan of the neck with contrast revealed a 4 cm × 8 cm × 3 cm cystic mass in the left neck occupying nodal levels 2–4 deep to the sternocleidomastoid muscle.

A 19-year-old male presented with a large left-sided neck mass that appeared suddenly during an upper respiratory infection (URI). Apart from noticing the mass, the patient was asymptomatic and denied pain or dyspnea. He did not have a history of prior neck masses, trauma, recent travel, smoking, or animal exposure. A computed tomography scan of the neck with contrast was completed and revealed a 4 cm × 8 cm × 3 cm cystic mass in the left neck occupying nodal levels 2–4 deep to the sternocleidomastoid muscle (Figure 1). The radiologic characteristics of the mass were thought to be consistent with an infected branchial cleft cyst. After reviewing the risks and benefits of surgery, the patient elected to undergo surgical excision for diagnostic and therapeutic purposes. In the operating room, the cyst was removed in its entirety without violation of the cystic capsule. All critical structures including the ipsilateral internal jugular vein, external and internal carotid arteries, vagus nerve, and phrenic nerve were identified and preserved. The patient was admitted for overnight observation and monitoring of drain output. The drain was removed on post-operative day one, and the patient was discharged home without issue. Final pathology was significant for a squamous cyst with adjacent thymus tissue, consistent with cervical thymic cyst. The patient's neck incision has healed appropriately, and he has not had any evidence of recurrence to date.

## 3. Discussion

A CTC is an uncommon clinical entity, and there are few small case series describing these lesions [1, 2]. A review of two pediatric patients with a CTC by De Caluwé and Ahmed in 2002 highlights the demographic characteristics associated with these lesions. CTCs most commonly occur in the pediatric population during the first decade of life [2]. According to data from Mikal in the 1970s, the median age at diagnosis is seven years [3]. These lesions are noted to be left-sided in the majority of patients, and males are twice as likely to be affected as females [4]. In a systematic review of the literature by Michalopoulos et al. in 2011, a total of 36 cases of a cervical thymic cyst occurring in an adult patient throughout history were identified [5]. Very few other reports of these lesions occurring in adults exist [6, 7]. Thus, our presentation of a patient with a clinically evident CTC at age 19 represents an uncommon presentation of an already rare clinical entity.

The origin of thymic cysts is not fully understood. The presence of asymptomatic thymic tissue in the neck is relatively common; however, a CTC is exceedingly rare. A thymic cyst can occur anywhere along the normal path of descent of the thymus from the mandible to the sternal notch. Various theories exist to explain the pathogenesis of these cysts. These entities may originate from degeneration of Hassall's bodies or possibly from remnants of the thymopharyngeal duct [2].

**Figure 2:**
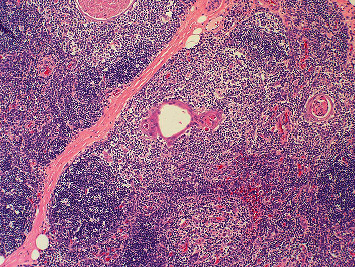
Hematoxylin and eosin, 20x. Thymic tissue, highlighting Hassall's corpuscles (central).

**Figure 3:**
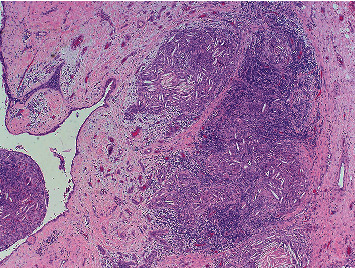
Hematoxylin and eosin, 4x. Cholesterol granuloma (right) with adjacent epithelial lined cyst (left).

Of the cervical thymic cysts reported, the most common presenting symptom is painless swelling in a child during the first decade of life. Upper airway symptoms such as dyspnea, dysphagia, hoarseness, and stridor have been reported in 6–13% of presentations [8]. Preoperative diagnosis of a CTC is challenging and there are no accepted radiologic criteria to identify these lesions. Definitive diagnosis depends on histologic analysis of the excised specimen. Gross evaluation of a CTC shows a soft, uniloculated or multiloculated mass which may or may not be accompanied by a tract [2]. Microscopic examination of the specimen will show thymic elements adjacent to the cyst cavity. These elements include Hassall's corpuscles, lymphocytic follicles, and thymic epithelial cells (Figure 2). Additionally, cholesterol granulomata are frequently observed (Figure 3) [9]. As with most cystic neck masses, the treatment of choice for these lesions is with definitive surgical excision. However, under appropriate circumstances, observation and sclerotherapy can also be considered as reasonable courses of action [10]. Surgery is often accomplished through a transverse neck incision, but a sternotomy may be required if there is thoracic extension of the lesion. Thymectomy during childhood is associated with impaired immune development, and thus, care must be taken to confirm the presence of a mediastinal thymus prior to excision of a presumed CTC [6]. Confirmation of a mediastinal thymus is not considered essential in adults prior to proceeding with surgery. Malignant degeneration is considered to be extremely uncommon for these lesions and has not been reported in a child [2].

## 4. Conclusion

The CTC is a rare benign cystic neck mass that most commonly presents in children during the first decade of life. Treatment of these cysts is with complete surgical excision, and the rate of recurrence is extremely low. Definitive diagnosis of these lesions depends on histologic examination of the excised specimen and can rarely be accurately diagnosed by preoperative imaging. We present the uncommon presentation of an adult male found to have a cervical thymic cyst. Although rare in clinical practice, the CTC should be kept in the differential diagnosis for cystic neck masses in both children and adults.
